# Can Prescribed Cannabinoids Substitute Cannabis?

**DOI:** 10.1192/j.eurpsy.2022.145

**Published:** 2022-09-01

**Authors:** A. Batalla

**Affiliations:** Utrecht UMC, Psychiatry, Utrecht, Netherlands

**Keywords:** medical cannabis, harm reduction, stigma, Cannabis

## Abstract

Legislative changes in the last years have made possible the prescription of medical cannabis in several countries, often following a growing public demand. However, the medical indications for use and the access to prescribed cannabis are still limited. Prescribers face several challenges in the form of barriers and dilemmas, often related to stigma, and deficient information and training. As a result, many people keep on using illicit cannabis for medical problems. In this session we will outline the most common controversies of cannabis prescription, particularly in psychiatry. We will discuss the ethical considerations regarding prescription practices, the benefit-risk assessment, the limitations of the current knowledge, and some potential solutions to respond to the strong demand from patients and families.

**Disclosure:**

No significant relationships.

